# Editorial: Deep learning in neuroimaging-based neurological disease analysis

**DOI:** 10.3389/fnimg.2023.1127719

**Published:** 2023-01-23

**Authors:** Xiaoxiao Li, Yu Zhang, Qingyu Zhao

**Affiliations:** ^1^Department of Electrical and Computer Engineering, The University of British Columbia, Vancouver, BC, Canada; ^2^Department of Bioengineering, Lehigh University, Bethlehem, PA, United States; ^3^Department of Electrical and Computer Engineering, Lehigh University, Bethlehem, PA, United States; ^4^Department of Psychiatry and Behavioral Sciences, Stanford University, Stanford, CA, United States

**Keywords:** neuroimaging, deep learning, neurological disease, machine learning, multimodality

Improving the understanding, prognosis, diagnosis, and treatment of neurological diseases has been increasingly relying on acquiring large-scale neuroimaging data from diverse participant groups to investigate altered brain structure and function linked to the diseases. One ongoing challenge in such neuroimaging studies is to establish scalable, sensitive, and robust computational approaches to disentangle disease-related information from high-dimensional, heterogeneous imaging data. To this end, deep neural networks, the state-of-the-art machine learning models, have become an emerging analytical tool. Compared to prior machine learning methods, deep learning methods have the advantage of reducing the need for manually engineering features from neuroimaging data, which traditionally relies on task-specific, simplified a priori knowledge. As such, deep learning has shown unprecedented power in clinical tasks including personalized treatment planning, disease progression forecasting, diagnosis classification, tumor localization, etc. However, there are also notable challenges associated with deep learning in neuroimaging studies. First, the sample size in typical neuroimaging studies is relatively small compared to the dimension of acquired imaging data, which often increases the chance of overfitting in the training of deep learning models. This data-scarce problem is exacerbated in multi-modal analysis (e.g., structural and functional MRI analysis), where study participants often have missing modalities. Moreover, it is generally not straightforward to reason about the decision process of a deep network. The lack of interpretability hinders the understanding of disease mechanisms and prevents the integration of deep learning tools in the clinical workflow. Herein, our Research Topic focuses on recent advances in the applications of deep learning to analyze neurological diseases based on neuroimaging data, which specifically deal with the challenges mentioned above.

A promising way to mitigate the small sample size problem is to leverage the concept of transfer learning, where deep networks can be pre-trained on a related task with sufficient training samples. For example, in the application of automatic cerebral microbleed segmentation, Dadar et al. argued that directly training a deep learning model on microbleeds data requires large-scale ground-truth manual segmentation, which is time-consuming to acquire and subject to inter-rater and intra-rater variability. Instead, they showed that by pre-training a segmentation network on the classification of cerebrospinal fluid vs. brain tissue (where a large number of ground-truth segmentation labels can be easily obtained), the resulting model can be used as the initialization for the microbleed segmentation network. By further suppressing false positive detections in a *post-hoc* manner, Dadar et al. showed this transfer learning scheme can generate accurate and robust microbleed segmentation and has the potential to improve the treatment of cerebrovascular and neurodegenerative diseases. However, in their experiments, the algorithm was trained on high-resolution multi-modal MRI data, which is not always available in many clinical settings. For example, Anctil-Robitaille et al. realized that the spatial resolution of typical diffusion-weighted imaging (DWI) is significantly lower than that of T1w MRI, so they aim to design a deep network to synthesize high-resolution diffusion data from structural MRI. To do so, a Cycle-GAN network was trained on a set of unpaired high-resolution T1w and low-resolution diffusion MRI. The network maps a high-resolution T1w to a high-resolution diffusion MRI, which on the one hand can be mapped back to the original T1 and, on the other hand, is not distinguishable from the real diffusion data after downsampling. In particular, the authors modeled the non-Euclidian properties of diffusion tensors using a Riemannian framework to make the generated diffusion MRIs physically plausible. Anctil-Robitaille et al. argued that this method could be used for missing modalities synthesis and datasets completion. Indeed, having complete multi-modal data is crucial for neurological disease analysis as they provide complementary information about the brain structure and function. This is also indicated in the work by Canalini et al. showing that the choice of MRI sequences has a huge impact on the registration accuracy of longitudinal data. Although the FLAIR sequence is useful for highlighting periventricular hyperintense lesions, such as multiple sclerosis (MS) plaques, it is the least informative sequence in the registration process, which heavily relies on the contrast enhanced T1w MRI and is influenced by the presence of pathology. Finally, Guo et al. focused on using deep learning to characterize brain function alterations associated with the progression of early-stage Parkinson's disease. They used a long-short-term memory network (LSTM) to distinguish patient cohorts based on time series data from resting-state function MRI. After achieving a significant classification accuracy, the learned LSTM model weights were used to select the top brain regions that contributed to model prediction, which characterized functional changes linked to motor impairment and gained better insight into the brain mechanisms of Parkinson's disease.

In summary, the above results published in this Research Topic addressed the challenges and underscored the great potential of deep learning methods to improve the analysis of neurological diseases using neuroimaging data. We foresee that further research on this topic will continuously focus on harmonizing heterogeneous multi-modal and longitudinal data to build unbiased deep learning models to advance scientific discoveries and improve clinical workflow.

## Author contributions

All authors listed have made a substantial, direct, and intellectual contribution to the work and approved it for publication.

